# Selection Signature Analysis of Whole-Genome Sequences to Identify Genome Differences Between Selected and Unselected Holstein Cattle

**DOI:** 10.3390/ani15152247

**Published:** 2025-07-31

**Authors:** Jiarui Cai, Liu Yang, Yahui Gao, George E. Liu, Yang Da, Li Ma

**Affiliations:** 1 Department of Animal and Avian Sciences, University of Maryland, College Park, MD 20742, USA; jiaruic@umd.edu (J.C.); lyang016@umd.edu (L.Y.); yahui.gao@scau.edu.cn (Y.G.); 2 Animal Genomics and Improvement Laboratory, Beltsville Agricultural Research Center, United States Department of Agriculture, Beltsville, MD 20705, USA; george.liu@usda.gov; 3 Department of Animal Science, University of Minnesota, St Paul, MN 55108, USA; yda@umn.edu

**Keywords:** Holstein cattle, selection signature, genome sequence, selection

## Abstract

A unique line of Holstein cattle has been maintained without selection in Minnesota since 1964. After many generations, unselected cattle produce less milk, but have better reproductive performance and health traits when compared with contemporary cows. Comparisons between this line of unselected Holstein and those under selection provide useful insights that connect selection and complex traits in cattle. Utilizing these unique resources and sequence data, we sought to identify genome changes due to selection. We sequenced 30 unselected and 54 selected Holstein cattle and compared their sequence variants to identify selection signatures. After many years, the two populations showed completely different patterns in their genome-level population structures and linkage disequilibrium. By integrating signals from five different detection methods, we detected consensus selection signatures from at least four methods covering 14,533 SNPs and 155 protein-coding genes. An integrated analysis of selection signatures with gene annotation, pathways, and the cattle QTL database demonstrated that the genomic regions under selection are related to milk productivity, health, and reproductive efficiency. The polygenic nature of these complex traits is evident from hundreds of selection signatures and candidate genes, suggesting that long-term artificial selection has acted on the whole genome rather than a few major genes. In summary, our study identified candidate selection signatures underlying phenotypic differences between unselected and selected Holstein cows and revealed insights into the genetic basis of complex traits in cattle.

## Simple Summary

Using a unique line of Holstein cattle unselected since 1964, we compared the genomes of unselected and selected Holstein cattle and reported genome differences due to long-term selection. We also integrated selection signatures with gene annotation, pathways, and the cattle QTL database to further explore the functional link between selection signatures and economic traits. The candidate selection signatures were involved in multiplex functions such as milk production, reproduction, and health. We confirmed that long-term artificial selection affected the whole genome rather than a few major genes due to the polygenic nature of the complex traits under selection.

## 1. Introduction

Over hundreds of years, selective breeding has greatly increased production and meanwhile changed the genomes of domestic animals [1]. In dairy farming, selection and breeding practices are routinely employed to enhance economic traits such as milk yield, protein and fat content, and reproductive efficiency [2]. More recently, the U.S. dairy industry has shifted selection goals to traits related to disease resistance and efficiency (https://uscdcb.com/). Over generations, such artificial selection has left distinctive imprints across the bovine genome, known as selection signatures, reflecting the genetic basis of economically important dairy traits.

In this study, we used two groups of Holstein cattle, a selected group and an unselected line under the same management and environment conditions at the University of Minnesota since 1964 [3]. The unselected line was bred within the group without any selection, and the selected group was bred with commercial bull semen with top milk production. Due to these different breeding strategies, the control line remained unchanged for most traits, while the selected group had an increasing trend as to the national average for production traits, but a decreasing trend for fertility traits. As the unselected and selected lines were maintained under the same conditions, we hypothesized that genome differences between the two lines are responsible for the divergent phenotypic variation. Understanding the genomic differences underlying these two different populations is crucial not only for mapping genes related to economic traits, but also for maintaining the genetic diversity and long-term sustainability of breeding programs [4].

Many selection signature analysis methods have been developed to detect genomic regions under selection that are sensitive to distinct time scales and types of selective sweeps. For instance, **population differentiation-based methods** such as the Fixation Index (Fst) detect distant selection events with strong interpopulation differentiation. Nucleotide diversity can be used to identify genomic regions that have significantly reduced nucleotide diversity [5]. **Haplotype-based methods** such as cross-population extended haplotype homozygosity (XP-EHH) is useful for ongoing or nearly fixed sweeps [6], and the integrated haplotype score (iHS) is sensitive to rapidly increasing derived allele frequencies [7]. **Allele frequency differentiation-based methods** like the cross-population composite likelihood ratio (XP-CLR) effectively detects differences in allele frequencies at multiple loci between two populations without relying on changes in population size [8]. Different methods capture different aspects of selection, but may also miss signals due to their distinct assumptions and sensitivities.

**Figure 1:**
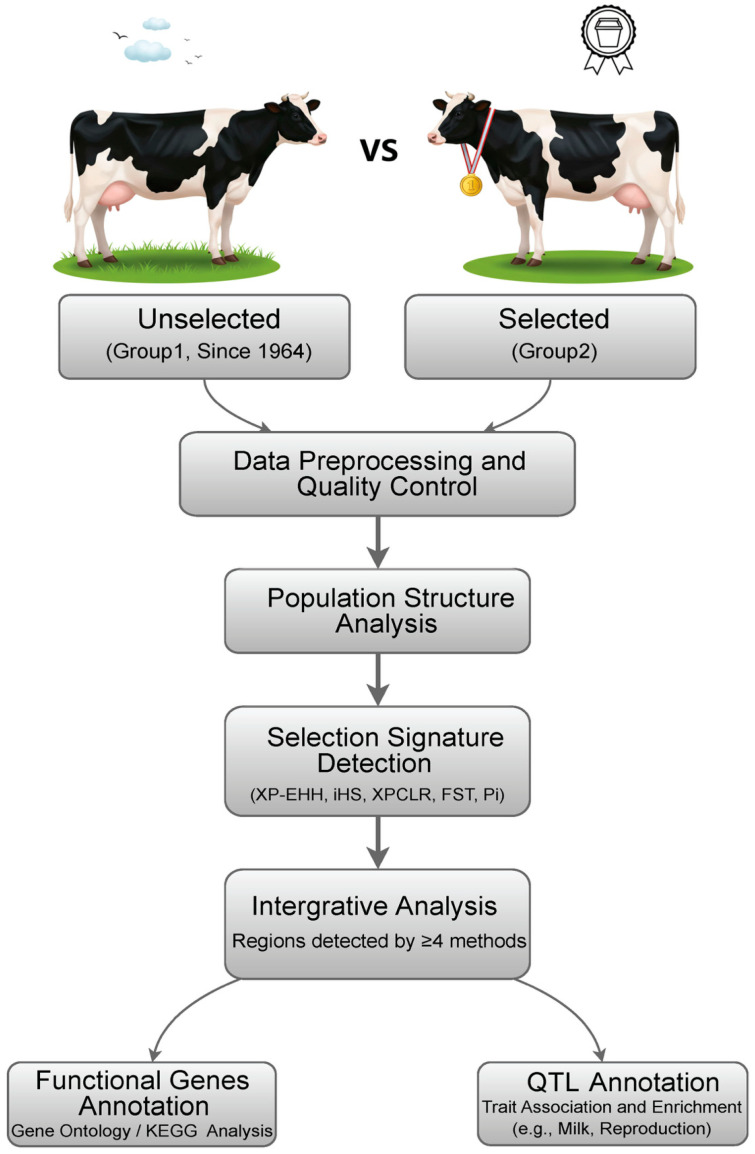
Overview of study design and analysis.

Previously, we reported genome differences between these two groups of cattle using 50K SNP array data [9], which have limited resolution. In this study (Figure 1), we conducted whole genome resequencing analysis on 84 Holstein cattle from the unselected (*n* = 30) and selected lines (*n* = 54). We carried out direct genomic comparisons using five methods for selective signature detection: XP-EHH, the iHS, the XP-CLR, the θ/π ratio, and the Fst. We also assembled 8285 quantitative trait loci (QTL) and 155 candidate genes covering six major trait types, especially those associated with milk production and reproduction traits. Employing a set of complementary allele frequency and haplotype-based methods, along with the annotation of QTLs and candidate genes, this study aimed to identify selection signatures underlying phenotypic differences between unselected and selected Holstein cows and to reveal a comprehensive understanding of the genetic basis of complex traits in cattle.

## 2. Materials and Methods

### 2.1. Study Population and Sample Collection

This study used two groups of unique Holstein cattle resources (Figure 1): a closed herd maintained without selection since 1964 from Minnesota (GP1, *n* = 30) and a long-term artificially selected Holstein population in the United States breeding system (GP2, *n* = 54) [9]. Blood samples were collected from all individuals, and whole-genome sequencing data were generated.

### 2.2. Whole Genome Sequencing and Data Quality Control

Tissue samples were processed and sequenced with standard procedures. Variants were called per-sample using the DeepVariant model with option “WGS” [10]. Quality control measures were applied to ensure high-quality single nucleotide polymorphism (SNP) datasets. All of the acquired SNP data were quality checked using Plink v1.90 software [11], including the elimination of SNPs with low quality (QUAL < 10), a low minor allele frequency (MAF < 0.05), a high missing rate (F_MISS > 0.5), and multiple allelic variants. Finally, a total of 12,381,574 high-quality bi-allelic SNPs were retained for downstream analysis.

### 2.3. Analysis of Diversity and Population Structure

To compare genetic diversity between the two groups, filtered high-quality SNPs were used to estimate observed and expected heterozygosity (H_o_ and H_e_, respectively) and the F coefficient with Plink v1.90. To evaluate population structure, filtered SNPs were first pruned for linkage disequilibrium (LD) in Plink v1.90 using the indep-pairwise command with parameters “50 5 0.2.” Specifically, a genome-wide scan was conducted with a 50-SNP window shifted by 5 SNPs at each step, and SNPs with an r^2^ of more than 0.2 were removed. Principal component analysis (PCA) was then conducted on the LD-pruned SNPs, and the results were visualized using the ggplot2 package in R [12]. To estimate and compare the patterns of LD between the unselected and selected populations, the LD coefficient (r^2^) was calculated for each pair of SNPs. With a maximum distance of 500 kb, PopLDdecay v3.4218 was used to generate LD decay plots based on the distance between SNPs [13].

### 2.4. Selection Signature Analysis

To carry out comprehensive analysis of significant genomic signals under selection, we employed five different detection methods: the fixation index (Fst), also known as the genetic differentiation index or Fst analysis; nucleotide diversity (PI); the cross-population composite likelihood ratio test (XP-CLR) [5,14]; cross-population extended haplotype homozygosity (XP-EHH) [6]; and the integrated haplotype score (iHS) to detect selective signatures between and within populations [7]. Each method was chosen for its ability to capture selection signals from different perspectives. All analyses were performed using standardized pipelines and software tools appropriate for each method. The Fst, the θ/π ratio, and allele frequencies were calculated using VCFtools with a 50 kb sliding window and a step size of 20 kb for scanning the whole genome [15]. The XP-CLR program used a 50 kb sliding window with a step size of 20 kb. XP-EHH and iHS scores were computed using Selscan with a 50 kb sliding window and a default step size with normalization applied to ensure comparability across genomic regions [16]. Finally, we summarized the common selection signatures within the top 5% thresholds in each method. To minimize false positives and enhance the reliability of detected signals, we focused on the top 5% of SNPs identified by at least four out of the five methods.

### 2.5. Functional Annotation and Enrichment Analysis

Annotation of reference genes was obtained from the ARS-UCD 2.0 bovine reference genome (gff_gcf_cattle_2.0) [17]. The significant SNPs were annotated and mapped to genes using the findOverlaps and queryHits functions in the GenomicRanges package [18]. Furthermore, candidate gene sets were identified and tested for enrichment using Shiny 0.82 for KEGG pathways [19] and the Metascape tool for GO biological process enrichment analysis [20].

### 2.6. Cattle QTL Annotation and Analysis

To further investigate the functional relevance of genomic regions under selection, QTL annotation was performed using the R package GALLO (v1.4.4) [21] with a ±250 kb window around each significant SNP to capture associated QTLs. Cattle QTL data were extracted from the Animal QTLdb (Animal_QTLdb_release55_cattleARS_UCD2.gff) [22]. Annotated QTLs were further categorized into functional groups, and functional enrichment analysis was conducted to explore potential biological pathways influenced by the identified selection signals.

## 3. Results

### 3.1. Population Analysis Identified Genome-Wide Differences Between Selected and Unselected Cattle

After quality control, a total of 12,381,574 high-quality bi-allelic SNPs were retained for population structure analysis. The average missing rate after quality control was 2.6%, indicating high genotyping reliability. To evaluate the genetic diversity of the population, we estimated the genome-wide inbreeding coefficients (F) of the two groups (Table 1), with the unselected population exhibiting a higher level of inbreeding (0.0783 ± 0.0155 SE) compared to the selected population (0.0493 ± 0.0095 SE). Correspondingly, the average observed heterozygosity (H_o_) ratio was 0.291 in the unselected group and 0.305 in the selected group, while the expected heterozygosity (H_e_) ratios were 0.316 and 0.321, respectively.

**Figure 2:**
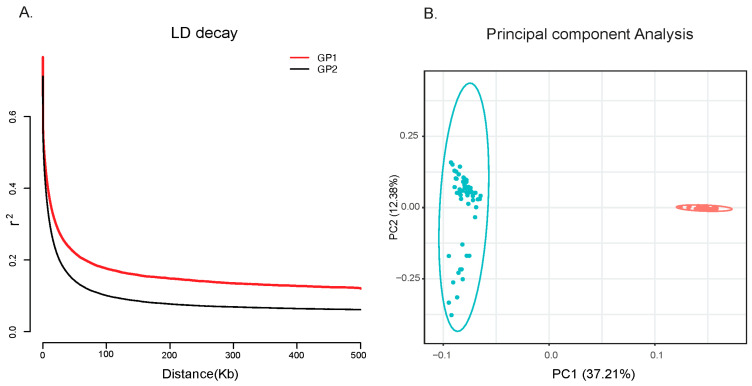
Population LD and structure of two groups of Holstein cattle. (**A**) Genome-wide LD decay estimated from each population, with x indicating the distance between SNPs and the *y*-axis representing the squared correlation (r^2^) between pairs of SNPs. (**B**) Principal component analysis of two cattle groups, with x and y axes representing principal components 1 and 2, respectively.

The LD decay analysis revealed that the unselected population exhibits a relatively slower LD decay rate (Figure 2A). Additionally, principal component analysis (PCA) using LD-pruned SNPs revealed a clear separation between the animals of these two groups (Figure 2B).

### 3.2. Selection Signature Analysis with Five Methods Identified Candidate Regions Under Selection

**Figure 3:**
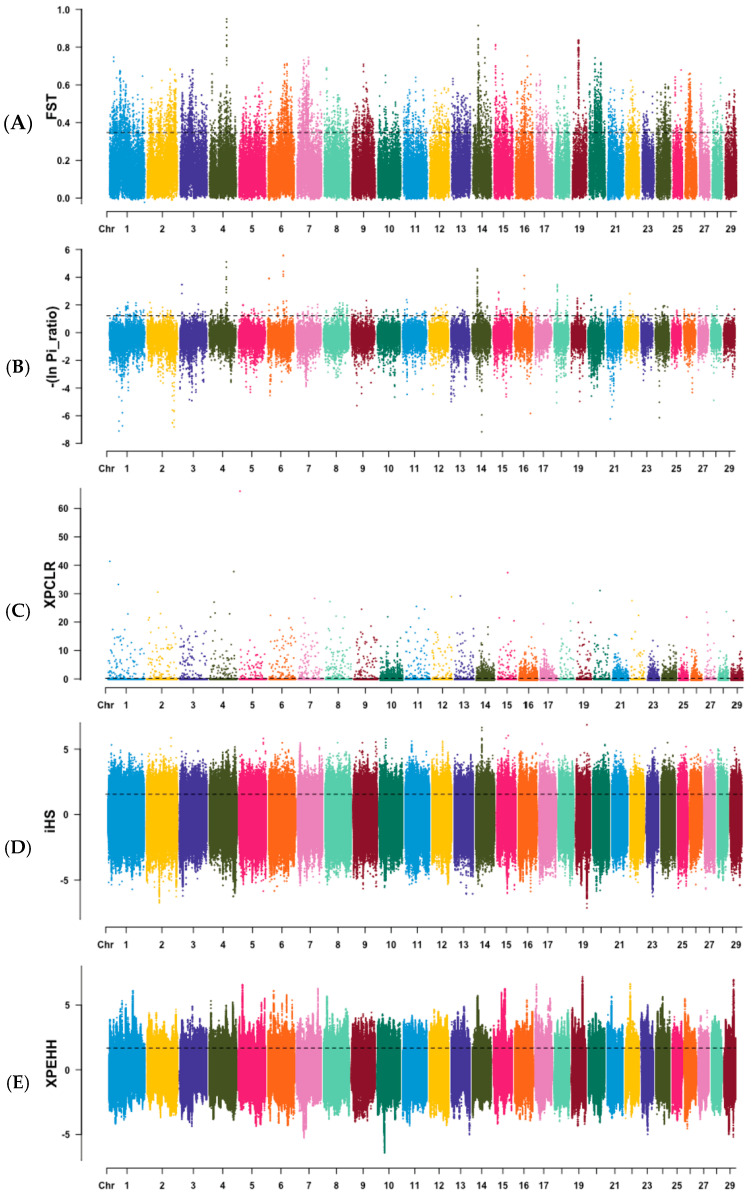
Manhattan plots of genome-wide selection signatures by five different methods. (**A**) Genome-wide distribution of Fst windows. (**B**) Genome-wide distribution of -ln(Pi_ratio) windows. (**C**) Genome-wide distribution of XP-CLR windows. (**D**) Genome-wide distribution of iHS values. (**E**) Genome-wide distribution of XP-EHH values. Black dashed lines represent the threshold of the top 5% values, and windows above the black lines were considered candidate regions of selection signatures.

Manhattan plots of five methods were used to show specific selection signatures from each method (Figure 3).

**Figure 4:**
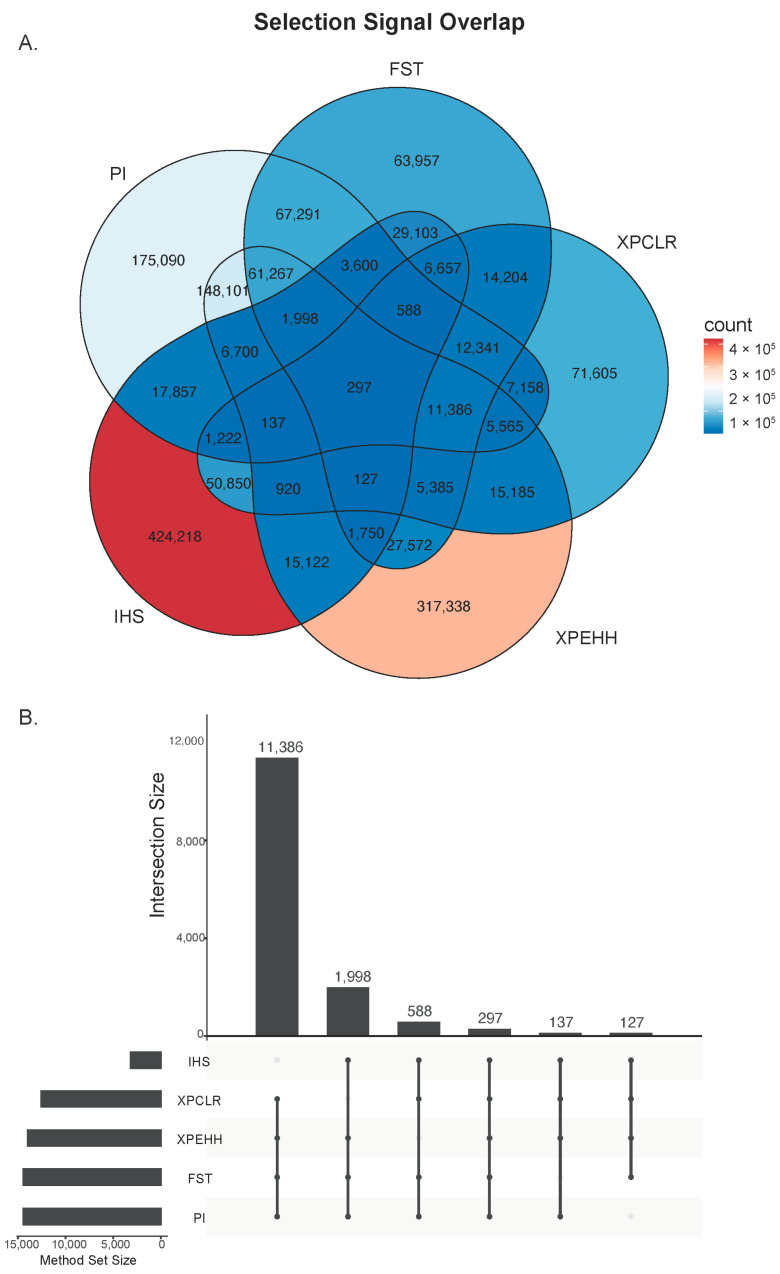
Identification of consensus selection signatures from four or more methods. (**A**) Venn diagram of candidate regions shared by five methods. (**B**) Number of candidate regions shared by four or five methods.

Genome-wide selection signature scanning by five methods (Fst, Pi, XP-CLR, XP-EHH, and iHS) showed a large proportion of overlap in the detected candidate regions among different methods (Figure 4A). A total of 14,533 SNPs were fully covered by four or more methods. Of the 14,533 SNPs supported by ≥4 statistics, around 21% of them mapped to BTA 14 (2975), 14% to BTA 16 (2092), and 11% to BTA 17 (1613). These candidate regions include genes *PLAG1* [23], *PREX2* [24], *CAPN2* [25], and *EDNRA* [26]. It is noteworthy that the iHS method, although it detected a higher number of signals, showed a lower percentage of overlap with other methods (Figure 4B).

### 3.3. Gene Annotation and Enrichment Analysis of Selection Signatures

**Figure 5:**
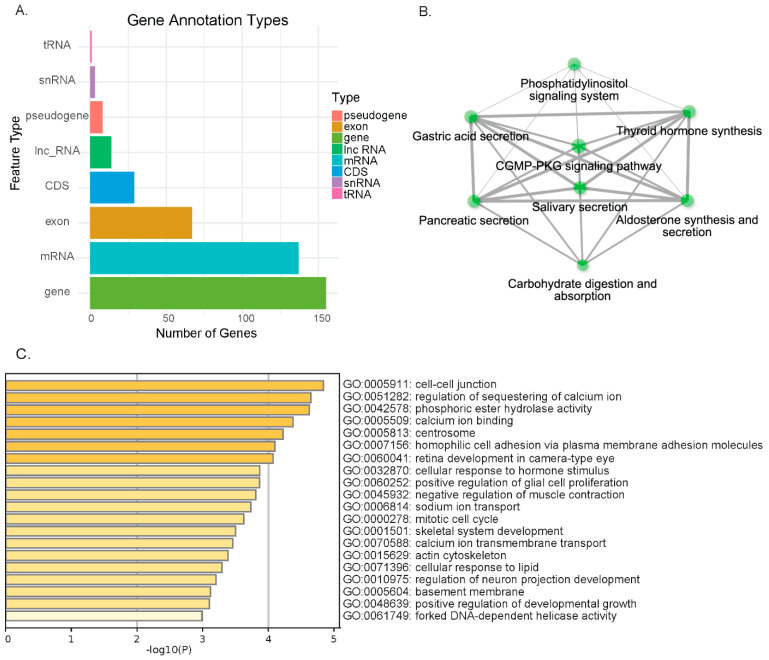
Gene and functional enrichment analysis of candidate selection signatures. (**A**) GO term enrichment for genes within candidate selection signatures. (**B**) Functional region proportion in overlapped genes. (**C**) KEGG term enrichment for genes within candidate regions.

A total of 163 genes were annotated in the candidate selection signature regions (top 5% from at least four methods) (Figure 5A). Among these, 155 genes had transcript-level annotations and were classified into 137 protein-coding genes and 14 long non-coding RNA (lncRNA) genes. Additionally, eight genes were annotated as pseudogenes, which were identified at the gene level but lacked corresponding transcript records.

GO and KEGG pathway enrichment analyses revealed significant processes related to milk production and overall physiological homeostasis. These included core metabolic functions and ion homeostasis, such as lipid and carbohydrate metabolism, and calcium and sodium ion transport (Figure 5B). Crucially, hormone-related pathways, including thyroid hormone synthesis and cellular response to hormone stimuli, were also enriched (Figure 5C). Furthermore, annotations highlighted processes vital for cellular structure, growth, and development, such as cell–cell junctions, skeletal system development, and mitotic cell-cycle regulation. Collectively, these enriched functions underpin the superior production performance and overall physiological resilience observed in the selected dairy cattle.

### 3.4. Cattle QTL Enrichment Analysis in Candidate Selection Signatures

**Figure 6:**
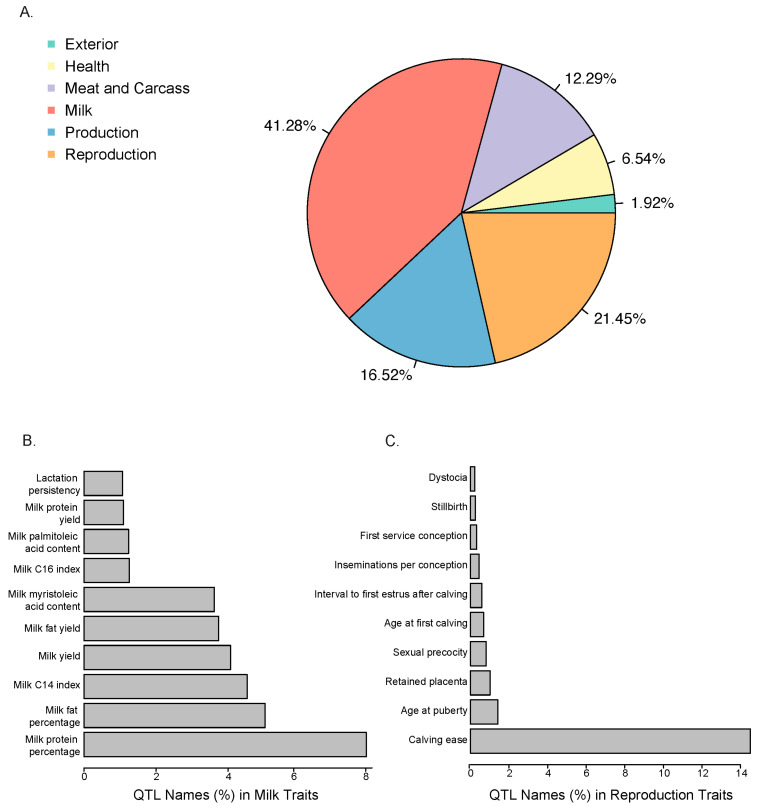
Proportional distribution of QTLs annotated from selection signatures across trait categories. (**A**) Percentage of QTLs for different trait categories. (**B**) Enrichment analysis results of QTLs in milk production traits. (**C**) Enrichment analysis results of QTLs in reproduction traits.

Using the cattle QTL database [22], we annotated QTLs within a 250 kb window around the significant selection signature SNPs to better understand their biological importance. An integrated analysis of 8285 annotated QTLs discovered six major trait categories (Figure 6A), indicating a strong enrichment for selection signatures associated with economically important traits. Notably, selection signatures were predominantly associated with milk production and reproduction traits.

Focusing on production-related traits, the top 10 annotated QTLs included traits related to milk composition and yield, milk protein percentage, milk fat percentage, milk yield, and milk fat yield (Figure 6B). Traits related to milk fatty acid profiles, including the C14 index, C16 index, palmitoleic acid content, and myristoleic acid content, were also notably enriched, suggesting selection pressures on both milk quantity and milk quality. Similarly, in the reproduction category (Figure 6C), annotated QTLs were mainly associated with key fertility and calving traits. Specifically, QTLs related to calving ease and age at puberty were predominant.

**Figure 7:**
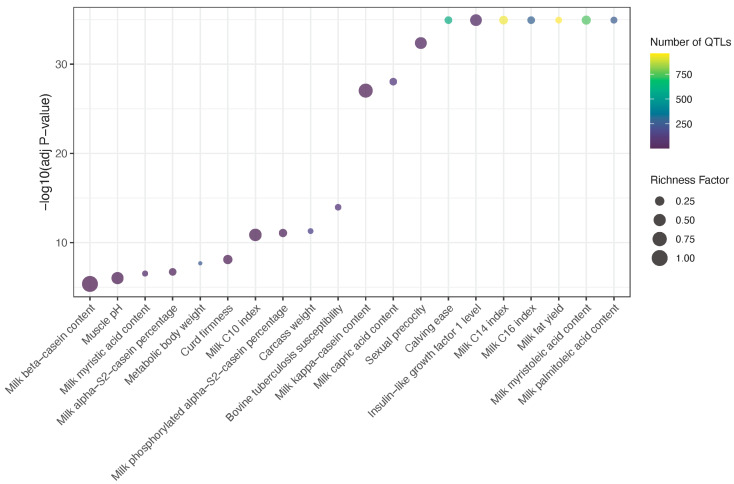
Top 20 enriched traits for QTLs near selection signature SNPs. Richness factors were obtained by calculating the ratio of the number of QTLs annotated in the candidate regions and the total number of QTLs.

The functional relevance of these identified selection signatures was further explored by functional enrichment analysis. Consistent with the QTL distribution in different types, these signatures are predominantly associated with a comprehensive suite of economically important traits, including various aspects of milk production and composition (e.g., milk fat yield, protein content, diverse fatty acid profiles, casein percentages, and rennet coagulation properties) directly aligning with the metabolic, hormone-related, and digestive pathways identified through gene enrichment (Figure 7). Furthermore, significant QTLs linked to reproductive performance (e.g., sexual precocity, calving ease, and dystocia) and growth and carcass characteristics (e.g., metabolic body weight, carcass length, and muscle PH) were also identified.

## 4. Discussion

This study employed an integrative analysis of selection signatures between a closed herd without selective breeding (unselected group) and contemporary commercially bred animals (selected group) and yielded a robust set of genomic and biological insights regarding selection. A total of 14,533 sequence variants were consistently identified by four or more selection signature methods, overlapping with 155 genes. This high concordance across different methods underscores the reliability of the detected selection signals, providing a strong foundation for understanding the genomic architecture shaped by artificial selection.

Comparison between the two groups of Holstein cattle on the genome-wide level suggests that the unselected population exhibits a slightly reduced level of heterozygosity and elevated inbreeding, likely due to its smaller effective population size and greater degree of local mating from historical isolation. In contrast, the selected group, representing a commercially selected population, shows higher heterozygosity and lower inbreeding, possibly reflecting structured breeding programs that maintained genetic variability.

Gene Ontology (GO) and KEGG pathway enrichment analyses of the 155 candidate genes revealed their involvement in a diverse array of biological processes that are critical for dairy cattle performance. Several biologically relevant terms were identified, possibly reflecting the physiological demands of intensive milk production, as well as the physiological processes of milk synthesis and secretion in dairy cattle [27]. Pathways related to cellular metabolism and ion homeostasis were significantly enriched, including those governing lipid metabolism (*CAPN2*, *LYN*, *PAX2*, *RET*, *PLCB1*, *PAQR8*, and *GPR155*). *CAPN2*, *LYN*, *PAX2*, and *RET* have established roles in mammary gland development and function related to milk production and post-lactational processes [28,29,30,31], and *PAX2* has been indicated to be relevant to milk fatty acid in Holstein cows [32].

The enrichment analysis identified another important pathway relevant to carbohydrate digestion and absorption and the transport of calcium and sodium ions (*EDNRA*, *PKD2L1*, *ITPR2*, *SLC17A1*, *SLC38A4*, *PTPRC*, *TRPV2*, *DST*, *CAPN2*, *MAN1A1*, *SCGN*, *MYOF*, *EFCAB2*, *ADGRE3*, and *EFHC1*). *ITPR2* has been identified as a significant gene associated with milk yield and myristic acid percentage in milk [33]. A previous study reported the association of *MAN1A1* with milk production and nitrogen excretion in dairy cows [34]. *TRPV2* has been reported as associated with resistance to mastitis as well as milk yield and milking rate [35,36]. Furthermore, genes and pathways related to hormone regulation and signaling were prominently identified, indicating the importance of endocrine systems on growth and reproduction, which are intensely selected in cattle breeding programs. For example, the *GHR* gene serves an essential role in reproductive function through mediating the influence of growth hormone (GH) in diverse reproductive events [37]; *EDNRA* and *SMYD3* are associated with reproductive traits in Holstein cattle [38]. *PAQR8*, a membrane progesterone receptor, was shown to have a possible role in mediating progesterone signaling, which is important for reproductive processes [39,40].

Additionally, the gene and enrichment analysis found pathways associated with cellular structure, growth, and development, including genes *ADD3*, *CTNNA2*, *PTPRM*, *PDZD2*, *KAZN*, *DNMBP*, *PLEKHA7*, *THEMIS*, *IGSF9*, *CNTN4*, *SDK1*, *LAMA1*, and *CADM2*. These genes and pathways are related to cell–cell junctions and homophilic cell adhesion, suggesting selection in dairy cattle for epithelial integrity and efficient cellular communication. Previously, studies have shown that *CTNNA2* (catenin alpha 2) is implicated in milk yield, milk fat, milk quality, and milking speed in Holstein cows [41,42]. *IGSF9, SDK1,* and *CADM2* are members of the immunoglobulin superfamily [43,44]. Their patterns of expression and functions imply a possible influence on tissue structure and milk production efficiency. Recent evidence suggests variants in *LAMA1* have been associated with improvements in genomic prediction accuracy for milk yield, highlighting *LAMA1* as a promising target for enhancing dairy cattle productivity [45].

The potential functional relevance of these selection signatures was also explored by an integrated analysis of 8285 cattle QTLs. These QTLs are associated with a comprehensive set of economically important traits, directly validating the importance of selection signatures and aligning with gene functions and pathways. Notably, dominant milk production QTLs might reflect the historical selection objectives aimed at improving dairy production and quality. QTLs for various aspects of milk composition (e.g., milk fat yield, protein content, diverse fatty acid profiles, casein percentages, and rennet coagulation properties) directly align with the identified metabolic, digestive, and hormone-related pathways. For instance, the enrichment of lipid metabolism genes (e.g., *FBP* and *GPR155*) and hormone signaling pathways provides a clear genetic basis for the observed improvements in milk fat and protein synthesis, as well as overall milk yield. Similarly, the prominence of reproduction-related QTLs (e.g., sexual precocity, lactation persistence, calving ease, and dystocia) might indicate breeding programs concurrently targeting functional traits to mitigate the fertility decline associated with intense selection for production. Along with enhancing economic returns, these could further reinforce the role of hormone signaling and developmental processes. For example, *GHR* is central to growth and metabolism. In addition, the presence of QTLs related to health and immune responses (e.g., interleukin-4 level and bovine tuberculosis susceptibility) further connects selection signatures to broader physiological adaptation, which could correspond to some pathways such as cell–cell junction and calcium ion binding, supported by genes like *LYN* [46] and *INPP4B* [47,48]. *INPP4B* facilitates cell survival and proliferation and supports the long-term lodgment of immune cells within tissues, which may highlight the potential of this gene as a high-value marker for genomic selection in dairy cattle for enhanced health and productivity.

## 5. Conclusions

Our results demonstrate that the genomic regions under selection in dairy cattle are critical for their superior productivity, health, and reproductive efficiency. The polygenic nature of these complex traits is evident from the hundreds of candidate genes and QTLs identified, which highlights that artificial selection has acted on the whole genome rather than on a few major genes. The candidate selection signatures provided useful insights into the genetic basis of complex traits and selection responses in dairy cattle. However, our study was limited by a small sample size and potential noises introduced by genetic drift. Therefore, in the future more powerful research is needed to elucidate how specific genetic variations affect economically important traits by combining selection signature results with functional association studies.

## Figures and Tables

**Table 1:** Genetic diversity of two Holstein populations.

Population	Observed Heterozygosity (H_o_)	Expected Heterozygosity (H_e_)	Inbreeding coefficient (F)
Unselected	0.291	0.316	0.078
Selected	0.305	0.321	0.049

## Data Availability

The whole-genome sequence data of the 30 unselected and 54 selected Holstein cattle were uploaded to the NCBI SRA database with SRA Bioproject number PRJNA1287090.
